# Optimization of a Virus-Induced Gene Silencing System with *Soybean yellow common mosaic virus* for Gene Function Studies in Soybeans

**DOI:** 10.5423/PPJ.OA.04.2015.0063

**Published:** 2016-04-01

**Authors:** Kil Hyun Kim, Seungmo Lim, Yang Jae Kang, Min Young Yoon, Moon Nam, Tae Hwan Jun, Min-Jung Seo, Seong-Bum Baek, Jeom-Ho Lee, Jung-Kyung Moon, Suk-Ha Lee, Su-Heon Lee, Hyoun-Sub Lim, Jae Sun Moon, Chang-Hwan Park

**Affiliations:** 1 National Institute of Crop Science, Rural Development Administration, Suwon 441-707, Korea; 2 Plant Systems Engineering Research Center, Korea Research Institute of Bioscience and Biotechnology, Daejeon 305-806, Korea; 3 Biosystems and Bioengineering Program, University of Science and Technology, Daejeon 305-350, Korea; 4 Department of Plant Science and Research Institute for Agriculture and Life Sciences, Seoul National University, Seoul 151-921, Korea; 5 School of Applied Biosciences, Kyungpook National University, Daegu 702-701, Korea; 6 Department of Plant Bioscience, College of Natural Resources & Life Science, Pusan National University, Pusan 627-706, Korea; 7 Department of Applied Biology, Chungnam National University, Daejeon 305-764, Korea

**Keywords:** *GmPDS*, optimal condition, soybean, SYCMV, VIGS

## Abstract

Virus-induced gene silencing (VIGS) is an effective tool for the study of soybean gene function. Successful VIGS depends on the interaction between virus spread and plant growth, which can be influenced by environmental conditions. Recently, we developed a new VIGS system derived from the *Soybean yellow common mosaic virus* (SYCMV). Here, we investigated several environmental and developmental factors to improve the efficiency of a SYCMV-based VIGS system to optimize the functional analysis of the soybean. Following SYCMV: *Glycine max*-phytoene desaturase (*GmPDS*) infiltration, we investigated the effect of photoperiod, inoculation time, concentration of *Agrobacterium* inoculm, and growth temperature on VIGS efficiency. In addition, the relative expression of *GmPDS* between non-silenced and silenced plants was measured by qRT-PCR. We found that gene silencing efficiency was highest at a photoperiod of 16/8 h (light/dark) at a growth temperature of approximately 27°C following syringe infiltration to unrolled unifoliolate leaves in cotyledon stage with a final SYCMV:*GmPDS* optimal density (OD)_600_ of 2.0. Using this optimized protocol, we achieved high efficiency of *GmPDS*-silencing in various soybean germplasms including cultivated and wild soybeans. We also confirmed that VIGS occurred in the entire plant, including the root, stem, leaves, and flowers, and could transmit *GmPDS* to other soybean germplasms via mechanical inoculation. This optimized protocol using a SYCMV-based VIGS system in the soybean should provide a fast and effective method to elucidate gene functions and for use in large-scale screening experiments.

The soybean (*Glycine max* (L.) Merr.) is an important legume crop. It is a major plant source of oil and provides protein-rich food for both human and animal consumption (Graham and Vance, 2003). Recently, a reference genome sequence of a cultivated soybean (*G. max* var. Williams 82) was released, and dozens of wild and cultivated soybeans have been resequenced (Kim et al., 2010; Lam et al., 2010; Schmutz et al., 2010). Furthermore, recent advances in next generation sequencing technologies have been applied to soybean research, leading to a large number of candidate genes. These genomic resources provide ample opportunities for elucidating gene functions involved in complex agronomical traits such as disease resistance, seed protein and oil content, and the number of seeds per pod in the soybean (Cook et al., 2012; Hwang et al., 2014; Pandy et al., 2011).

Reverse genetics has been used for plant functional studies and typically involves various methods and tools, including gene silencing, overexpression, T-DNA tagging, transposon tagging, zinc-finger nucleases, homologous recombination, Deletagene, and the targeting of local lesions in the genome (Anai, 2012). Nevertheless, transformation represents the most invaluable tool for functional studies because it allows for the production of novel and genetically diverse genotypes. Stable genetic transformation is not yet considered routine in the soybean due to several limitations to soybean transformation. Only limited cultivars, such as Jack, Williams 82, Throne, and Bert can be transformed (Yamada et al., 2012). Moreover, transformation efficiency is low, and the construction of transformed soybeans requires long periods of time (over 1 year) (Mano et al., 2014).

Virus-induced gene silencing (VIGS) is an attractive alternative for elucidating gene function in soybean without the need for stable transformation (Burch-Smith et al., 2004). VIGS has been widely used to silence target genes of interest through plant natural defense mechanisms (Lu et al., 2003). VIGS involves the delivery of a recombinant virus to plants containing a fragment of the plant gene that is intended to be silenced. The plant defense mechanism system then decreases not only the virus but also the targeted endogenous plant gene expression through post-transcriptional gene silencing (Robertson, 2004). Many viral vectors, both RNA and DNA, have been developed for gene silencing analysis. These viruses include *Tobacco mosaic virus*, *Potato virus X*, *Tobacco rattle virus*, *Barley stripe mosaic virus*, *Pea early browning virus*, *Poplar mosaic virus*, *Brome mosaic virus*, *Cabbage leaf curl virus, African cassava mosaic virus*, and *Tomato yellow leaf curl China virus* (Robertson, 2004; Unver and Budak, 2009).

However, only a few VIGS vectors are now available for soybean research based on the *Bean pod mottle virus* (BPMV) (Zhang and Ghabrial, 2006), *Cucumber mosaic virus* (CMV) (Nagamatsu et al., 2007), and *Apple latent spherical virus* (ALSV) (Yamagishi and Yoshikawa, 2009). Based on USDA soybean germplasm collection, there exist more than 14,000 accessions including wild, landrace, cultivated, and introduced soybeans (Bandillo et al., 2015). These VIGS vectors may not be able to be introduced into some soybean accessions, because some cultivars might have resistant genes against these viruses. Furthermore, ALSV and BPMV are not found in Korea and cannot be used as VIGS vectors in this area, necessitating new and more appropriate virus vectors to be developed for VIGS-based functional analyses of the soybean. Recently, Lim et al. (2015) developed a new VIGS system based on the *Soybean yellow common mosaic virus* (SYCMV) (Nam et al., 2012). Unlike ALSV, BPMV, and CMV consisting of bi- or tri-partite RNA genomes, SYCMV has a single stranded RNA genome. SYCMV-derived VIGS system is more efficient than other systems for developing VIGS constructs easily and rapidly.

For determining the effectiveness of VIGS, growth conditions, including photoperiods and the developmental stage of the plants at the time of inoculation, were cited as the most important factors (Burch-Smith et al., 2006). In addition, the VIGS vector, strain, culture concentration, inoculation method, photoperiod, plant age, and plant growth temperature have been highlighted as key parameters for VIGS effectiveness (Burch-Smith et al., 2004; Wang et al., 2006). Therefore, much effort has been invested to ascertain the optimal conditions for VIGS in plants (Pang et al., 2013; Sung et al., 2014; Wang et al., 2013).

The aim of this study was to determine the optimal conditions for the SYCMV-based VIGS system through investigation of several factors that potentially influence VIGS efficiency. This study demonstrates that a new SYCMV-based VIGS system could be useful for a wide range of applications in soybean functional genomics.

## Materials and Methods

### Plant materials

Eight cultivated (Alchangkong, Danbaegkong, Jangmikong, Jangsukong, Jangyeobkong, SS2-2, Taekwangkong, and Williams 82) and eight wild (GWS 0030, GWS 0210, GWS 0368, GWS 0972, GWS 0999, GWS 1009, GWS 1237, and IT 182932) soybean germplasms (Kim et al., 2014) were used in this study. Soybean germplasm were germinated and grown in Wagner pots containing a steam-sterilized soil mixture. Seedlings from the cotyledon expansion to fourth trifoliolate emergence were used for the VIGS assays. The growth chamber conditions were maintained at 18–30°C under 8/16h, 12/12h, or 16/8 h light/dark photoperiods with 60% relative humidity.

### Construction of a SYCMV-derived VIGS vector and *Agrobacterium* infiltration

The SYCMV VIGS vector (WO2013154233A1) was provided by Dr. Moon of the Korea Research Institute of Bioscience and Biotechnology. To develop a SYCMV-derived VIGS vector, we constructed a 35S promoter-driven SYCMV clone based on a T7 promoter-driven SYCMV clone (Nam et al., 2012). The pPZP211 binary vector was used, and a *BsrGI* restriction enzyme site was generated for the cloning of the foreign sequence. Initially, the complete sequence of SYCMV was inserted into the pPZP211 binary vector flanked by a CaMV 35S promoter at the 5′ terminus of the SYCMV genome and a *cis*-cleaving ribozyme sequence (Rz) followed by a nopaline synthase terminator (NOSt) at the 3′ terminus of the SYCMV genome described by Lim et al. (2015). Additionally, we performed a single nucleotide substitution from A to C at position 4,040 nt in the SYCMV sequence to generate a *BsrG*I site at the 3′ UTR region 10 bp from the stop codon of the coat protein (CP) to allow for the simple insertion of an endogenous plant DNA fragment for VIGS. The 3′ UTR is an optimal region for this insertion because SYCMV has 4 putative ORFs that overlap with one another to strategically express viral products. Subsequently, the gene of interest was inserted into the *BsrGI* recognition site placed at the 3′ UTR.

A 207-bp fragment of *G. max* phytoene desaturase (*GmPDS*; GenBank accession no. M64704.1) was amplified by RT-PCR using two primers containing the *BsrGI* site (forward: 5′-AAATGTACATAACTGGAAAGGGATTCCATAT-3′ and reverse: 5′-AAATGTACAACCAACTCTAACATTGACTGGTT-3′) from the soybean cv. Jangyeobkong. The PCR-amplified fragment was cloned into a *BsrGI* site of the SYCMV vector in a sense orientation to produce SYCMV:*GmPDS*. Plasmids were then transformed into *Agrobacterium tumefaciens* strain GV3101 via heat shock (Panja et al., 2008). Agrobacterium cultures were grown overnight at 28°C in Luria-Bertani (LB) medium containing antibiotics (100 μg/ml spectinomycin and 50 μg/ml rifampicin). Cultured solution was then centrifuged and resuspended in 10 mM 2-(N-morpholino) ethanesulfonic acid (MES; pH 5.6), 10 mM MgCl_2_, and 200 μM acetosyringone. Cell suspensions were incubated at room temperature for at least 2 h without shaking and then SYCMV:*GmPDS* was infiltrated into two fully expanded cotyledons and primary leaves using a needle-less 1 ml syringe.

### Optimization of conditions for a SYCMV-based VIGS system in soybean

First, SYCMV:*GmPDS* constructs were infiltrated onto the primary leaves in plants and these filtrated plants were grown under three different photoperiods (16/8, 12/12, and 8/16 h [day/night]) to evaluate the effect of *GmPDS*-silencing. Second, SYCMV:*GmPDS* constructs were infiltrated at five different stages (cotyledon, unrolled unifoliolate, first trifoliolate, second trifoliolate, and third trifoliolate leaves) to investigate the effect of plant growth stage on photobleaching due to knock down of *GmPDS*. Third, SYCMV:*GmPDS* constructs were infiltrated onto the cotyledon and primary leaves at six different culture concentrations (OD_600_ = 0.5, 0.8, 1.0, 1.5, 2.0, and 2.5) at 25°C with a 16/8 h light/dark photoperiod. Lastly, a screening experiment was performed with sixteen soybean germplasm including *G. max* and *G. soja* to ascertain the appropriate soybean lines for the SYCMV-based VIGS system.

### Statistical analysis

VIGS efficiency analysis was conducted in triplicate with 10 plants per experiment. The phenotypic evaluation of gene silencing was calculated with the following formula: gene silencing efficiency (%) = (number of plants showing photobleaching phenotype/total number of tested plants) × 100% (Wang et al., 2013). The data was analyzed using the PROC GLM procedure of SAS 9.4 (SAS Institute Inc., Cary, NC, USA), and means of the different treatments were separated by least significant difference (LSD) test at a 5% significance level. Values are represented as means ± SD of three independent experiments.

### RNA extraction and quantitative real-time (qRT) PCR analysis

Total RNA from the leaves of SYCMV:*GmPDS*-infiltrated plants was extracted with the RNeasy Plant Mini Kit (Qiagen, USA), and cDNA was synthesized from 1 μg of total RNA using the iScript cDNA Synthesis Kit (Bio-Rad, USA), according to the manufacturers’ instructions. All samples were analyzed by PCR using Prime Taq premix (2×) (GeNetBio, Korea) and primer sets specific to SYCMV (forward: 5′-TTGGCTGAGAGGAGTGGCTT-3′ and reverse: 5′-TGCGGTCGTGTAGTCAGTG-3′) and *GmPDS* (forward: 5′-TTGCTGCATGGAAAGACAAG-3′ and reverse: 5′-ATAGCTGGGAGAAGCCCAAT-3′). In order to further quantify *GmPDS* gene expression among the healthy, SYCMV:empty, and SYCMV:*GmPDS* plants, qRT-PCR was performed with the Rotor-Gene™ 6000 system (Corbett Life Science, Australia) with iQ™ SYBR^®^ Green Supermix (Bio-Rad). The expression levels of the target genes were normalized to *G. max* tubulin (GenBank accession no. XM_003550379) expression. The initial denaturation step at 95°C for 10min was followed by 40 cycles at 95°C for 10 sec, 58°C for 20 sec and 72°C for 1 min. All samples were analyzed in triplicate to ensure statistical significance. Relative mRNA expression of *GmPDS* was measured with the 2-^(ΔΔCt)^ method (Schmittgen and Livak, 2008), using *G. max* tubulin as the reference gene.

## Results

### Effect of different cultivars on the SYCMV-based VIGS system

Cultivars of the same species could vary in their susceptibility to ALSV, BPMV, CMV, or *Soybean mosaic virus* (SMV) infection and their VIGS efficiency (Chiuoaru et al., 2012; Kandoth et al., 2013; Kawai et al., 2014; Soto-arias and Munkvold, 2011). To estimate the efficiency of SYCMV-based VIGS in different cultivars of the soybean, we examined gene silencing of the three elite cultivars; Jangmikong, Jangyeobkong, and Taekwangkong (Supplementary Fig. 1). We initially constructed SYCMV:*GmPDS* and tested its ability to suppress the expression of endogenous *GmPDS* in the soybean using the SYCMV-derived VIGS vector provided by the Dr. Moon of Korea Research Institute of Bioscience and Biotechnology. The *GmPDS*-silencing causes the suppression of carotenoid biosynthesis resulting in photobleaching symptoms in the silenced plants.

**Fig. 1:**
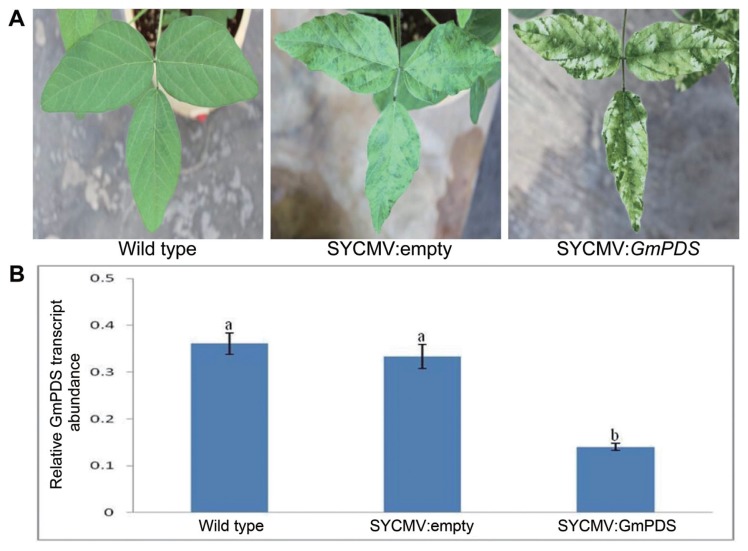
Phenotypic silencing and transcript accumulation of *GmPDS* in a SYCMV-based VIGS system under natural conditions. (A) Phenotypes of wild type, SYCMV:empty, and SYCMV:*GmPDS* vectors infiltrated with OD_600_ = 0.8 in the soybean cultivar, Jangyeobkong. The photographs were taken 30 days after the inoculation. (B) Real-time RT-PCR analysis of *GmPDS* gene expression among wild type, SYCMV:empty, and SYCMV:*GmPDS*-infiltrated plants. Error bars represent standard deviations (n = three biological replicates). Letters indicated significant differences using the LSD test at *P* ≤ 0.05.

This SYCMV:*GmPDS* inoculum was infiltrated at the primary leaves with a final OD_600_ of 0.8 in a greenhouse under natural conditions. Photobleaching was observed on the infected leaves approximately 30 days after inoculation (DAI). The efficiency of *GmPDS* silencing in the different soybean cultivars was shown in Supplementary figure 1. Among the three soybean cultivars, Jangyeobkong exhibited the most extensive *GmPDS*-silencing phenotype and had the highest observed gene silencing efficiency (73%), while the gene silencing efficiencies of Jangmikong and Taekwangkong were less than 60% (Supplementary Fig. 1). Plants infiltrated with the SYCMV:empty vector were used as controls in this study and showed the typical SYCMV symptoms of yellowing and mosaic formation (Fig. 1A). *GmPDS*-silencing at the molecular level was confirmed through qRT-PCR to evaluate the accumulation of the *GmPDS* gene transcript in leaves among wild type, SYCMV:empty, and SYCMV:*GmPDS*-infiltrated plants (Fig. 1B). There were no huge significant differences in the *GmPDS* mRNA expression in wild type and SYCMV:empty infiltrated plants. However, the *GmPDS* mRNA quantities were dramatically reduced in SYCMV:*GmPDS*-infiltrated plants with photobleaching symptoms. Based on these results, cv. Jangyeobkong was used as the target plant for optimization of SYCMV-based VIGS system in this study.

### Optimal conditions for an efficient SYCMV-based VIGS system in soybean

**Fig. 2:**
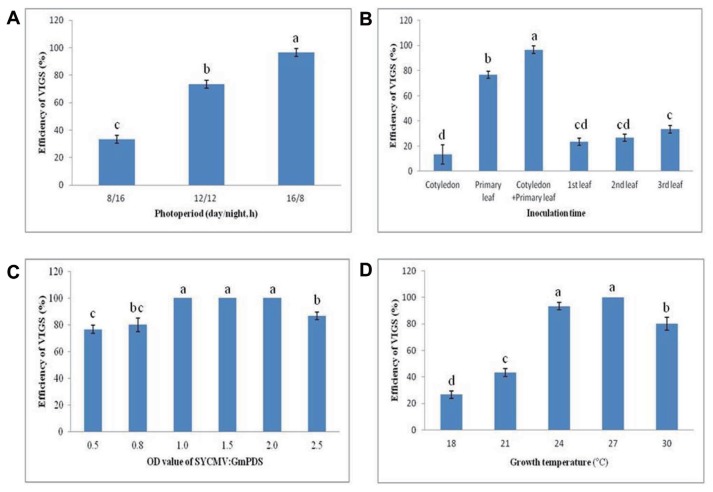
Optimal factors for an efficient SYCMV-based VIGS system in soybeans. (A–D) The percentage of plants showing photobleaching symptoms was affected by the photoperiod, inoculation time at growth stage, OD value of SYCMV:*GmPDS*, and growth temperature. All experiments were performed with ten independent biological replicates. Letters indicated significant differences using the LSD test at *P* ≤ 0.05.

We investigated the post-inoculation responses of the soybean under various conditions to optimize the efficiency of SYCMV-based VIGS vector (Fig. 2). Successful gene silencing depends on environmental and developmental factors including photoperiod, plant age, culture concentration, plant growth temperature, and inoculation method (Senthil-Kumar and Mysore, 2011; Wang et al., 2013). First, we compared the number of plants exhibiting photobleaching phenotype after SYCMV:*GmPDS* infiltration under different photoperiods (Fig. 2A). The number of *GmPDS*-silenced plants showing photobleaching symptoms when grown under long day (LD) conditions (16/8 h) was significantly higher than those grown under short day conditions (8/16 h). Under LD conditions, we observed the photobleaching of the infected leaves at approximately 20 DAI. All infiltrated plants displayed photobleaching symptoms under a 16/8 h photoperiod, compared to 70% and 20% under 12/12 h and 8/16 h photoperiods, respectively.

Next, we tested the effect of inoculation at different growth stages on VIGS efficiency under LD conditions (Fig. 2B). After each inoculation from cotyledon to the third trifoliolate stage, we observed photobleaching phenotypes at 25 DAI. Infiltration at the cotyledon and primary leaves exhibited the highest gene silencing efficiency of 100%. Although infiltration at the primary leaves exhibited a gene silencing efficiency of 80%, the efficiency at other growth stages was less than 30%. Therefore, infiltration at the cotyledon and primary leaves was considered optimal for the SYCMV-based system in soybeans.

In order to determine the optimal agroinfiltration concentration, different concentrations of *Agrobacterium* inocula (OD_600_ values of 0.5, 0.8, 1.0, 1.5, 2.0, and 2.5) were infiltrated at the cotyledons and primary leaves under LD conditions (Fig. 2C). Although there were no significant differences between OD_600_ values, the highest gene silencing efficiencies were observed in infiltrated plants with final OD_600_ of 1.0, 1.5, and 2.0. We also investigated the influence of the growth temperature of agroinfiltrated plants on gene silencing (Fig. 2D). After the infiltration of SYCMV:*GmPDS* inoculum with a final OD_600_ of 1.0, plants were grown in growth chambers at different temperatures (18°C, 21°C, 24°C, 27°C, and 30°C) under LD conditions. The SYCMV:*GmPDS*-infiltrated plants grown at 24°C and 27°C showed the highest gene silencing efficiencies of 90% and 100%, respectively. However, plants showing photobleaching were strongly inhibited at 18°C.

These results demonstrated that the SYCMV:*GmPDS*-silenced plants infiltrated at cotyledons and primary leaves grown at 24°C–27°C under LD conditions displayed photobleaching phenotypes with nearly 100% efficiency.

### Applications of a SYCMV-based VIGS system in various organs

**Fig. 3:**
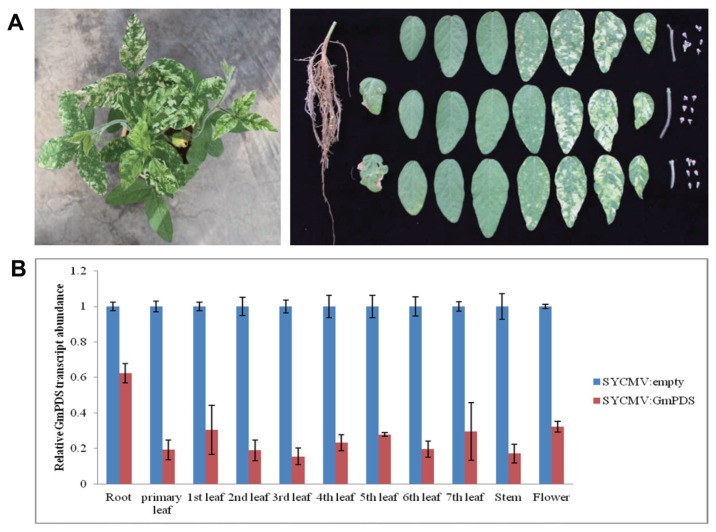
The efficacy of SYCMV-based VIGS in different soybean organs. (A) Phenotype of cv. Jangyeobkong 40 days after SYCMV:*GmPDS* infiltration (leaf) and morphology of different organs at that time. (B) qRT-PCR analysis of transcript abundance of *GmPDS* in different organs of SYCMV:*GmPDS*-infiltrated plants compared with SYCMV:empty-infiltrated plants. All experiments were performed with three independent biological replicates.

In order to test the practical usage of a SYCMV-based VIGS system in various plant organs, we investigated the ability of this VIGS system to spread throughout the entire plants including roots, leaves, stems, and flowers (Fig. 3). These samples were collected at 40 days after SYCMV:*GmPDS* infiltration when photobleaching was obvious in leaves and some flowers were in bloom each plant (Fig. 3A). The most extensive photobleaching was seen after the fifth leaves, whereas there was no significant differences in root, the primary, first, second, and third leaves, stems, and flowers between SYCMV:empty and SYCMV:*GmPDS*-infiltrated plants. SYCMV:*GmPDS*-induced gene silencing occurred in all organs tested (Fig. 3B). *GmPDS* transcript level was only moderately down-regulated in roots and the first trifoliolate, whereas it was strongly down-regulated in most of leaves, stems, and flowers in the silenced plants. Additionally, we evaluated seed coat morphology among mock, SYCMV:empty and SYCMV:*GmPDS*-inoculated plants (Supplementary Fig. 2). No seed coat mottling appeared in the healthy plants, but the seed mottling phenotypes observed in both SYCMV:empty and SYCMV:*GmPDS* inoculated plants. These results demonstrated that SYCMV-based VIGS system spread throughout the entire plants.

### Screening of soybean germplasms for efficient SYCMV-based VIGS response

**Fig. 4:**
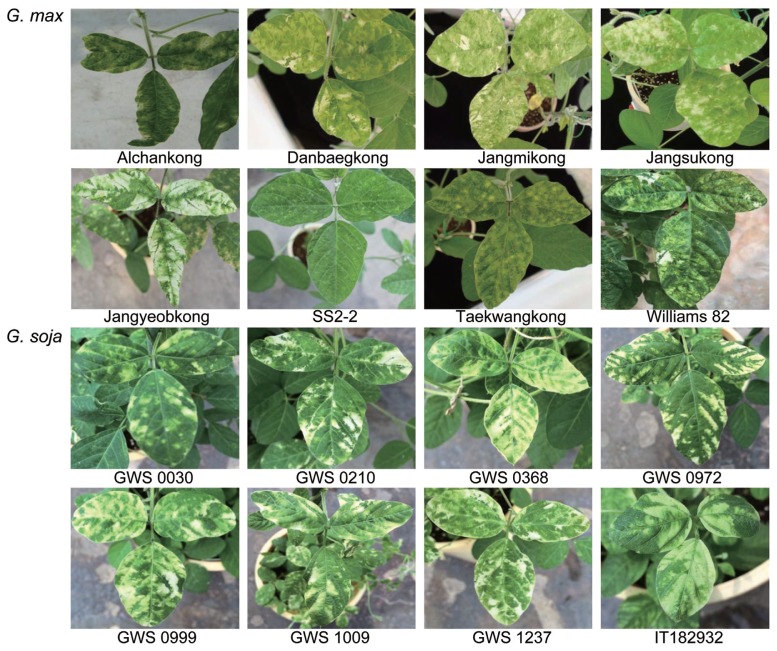
Phenotypes of sixteen *G. max* and *G. soja* after SYCMV-induced *GmPDS* silencing with syringe infiltration. A total of eight cultivated and eight wild soybean germplasm were observed after the SYCMV:*GmPDS* inoculation. All soybeans except cv. SS2-2 displayed a photobleaching phenotype. All leaves were photographed 25 days after inoculation.

In order to confirm whether the appropriateness of this SYCMV-based VIGS system protocol was appropriate for soybean germplasms, we performed a screening experiment using eight cultivated and eight wild soybeans (Fig. 4). As amended by the above-mentioned protocol, SYCMV:*GmPDS* inoculum with an OD_600_ of 1.0 was infiltrated onto the cotyledons and primary leaves of ten-day-old soybeans after planting ten seeds per genotype. SYCMV:*GmPDS*-inoculated plants were grown at 27°C under LD conditions. Of sixteen soybean germplasms, seven cultivated and eight wild exhibited photobleaching symptoms at 25 DAI. Among these, two cultivated soybeans, Jangmikong and Jangyeobkong, and three wild soybeans, GWS 0368, GWS 0999, and IT 182932, exhibited the most intense *GmPDS*-silencing phenotypes in all individuals (10/10). Williams 82 and IT 182932, which were completely sequenced (Kim et al., 2010; Schmutz et al., 2010), also showed obvious photobleaching symptoms. However, SS2-2 did not show obvious photobleaching induced by *GmPDS*-silencing (Fig. 4). Although syringe injection onto the primary leaves at the cotyledon stage was also tested, soybean cv. SS2-2 applied by the optimal protocol failed to produce results as consistent as other soybean germplasms; only three of ten individuals showed weak silencing of *GmPDS*.

**Fig. 5:**
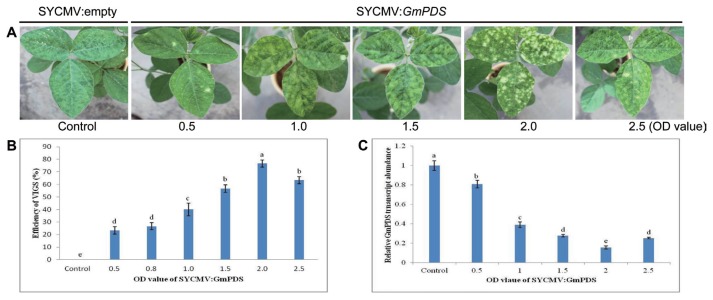
The effect of different concentrations of inoculum on the SYCMV-based VIGS efficiency in soybeans. (A) Phenotypes of soybean cv. SS2-2 infiltrated with five different concentrations. (B) The percentage of plants with photobleaching symptoms was affected by the OD value of SYCMV:*GmPDS*. All experiments were performed with ten independent biological replicates. Letters indicated significant differences using the LSD test at *P* ≤ 0.05. (C) The relative expression of *GmPDS* in SS2-2 leaves infiltrated with SYCMV:empty or SYCMV:*GmPDS* inoculum at five different concentrations.

Soybean cultivar SS2-2 induces the overexpression of jasmonate-responsive genes (Seo et al., 2007), resulting in a tolerance response to abiotic and biotic stresses (Kim et al., 2011). In order to address this problem with soybean cv. SS2-2 showing weak gene silencing, we adjusted the concentration of SYCMV:*GmPDS* inoculums (Fig. 5) based upon soybean cv. Jangyeobkong, which exhibited the highest gene silencing efficiency at OD_600_ = 1.0, 1.5, and 2.0 (Fig. 2C). We infiltrated cv. SS2-2 with five different concentrations (OD_600_ = 0.5, 1.0, 1.5, 2.0, and 2.5; Fig. 5A) and assessed the plants for photobleaching. We found that the plants infiltrated at a final OD_600_ of 2.0 showed obvious photobleaching symptoms (80%) induced by *GmPDS*-silencing (Fig. 5B), which was visually observed after the sixth trifoliolate, leading to a late photobleaching response at 35 to 40 DAI. *GmPDS* transcript accumulation was then evaluated through qRT-PCR on the sixth and seventh leaves showing photobleaching symptoms (Fig. 5C). We found that the *GmPDS* transcript level was dramatically down-regulated at a final OD_600_ of 2.0. Although this concentration did not result in 100% photobleaching in cv. SS2-2, we were able to achieve 80% photobleaching.

## Discussion

The completion of the whole genome sequence of the soybean (Kim et al., 2010; Schmutz et al., 2010) has allowed for the prediction of gene function and suggested candidate genes for complex agronomic traits. VIGS has been used as a fast and easy tool to assess gene function in plants; the BPMV-based VIGS vector is widely used for gene function study of rust and cyst nematode resistance in soybeans (Kandoth et al., 2013; Pandey et al., 2011; Zhang et al., 2010). Though the BPMV-based VIGS system is frequently used for gene function study, more VIGS vectors are needed for more broad application in numerous soybean germplasms. Recently, we developed a new SYCMV-based VIGS system (Lim et al., 2015). Accumulation of SYCMV RNA in SYCMV:*GmPDS*-infiltrated plant was relatively lower than that SYCMV:empty plant as a control. Also, silencing of *PDS* gene did not correlate with *Barley stripe mosaic virus* accumulation (Bruun-Rasmussen et al., 2007). Viral accumulation and VIGS efficiency are by nature dependent on the viral life cycle as well as interaction between plant and virus (Senthil-Kumar and Mysore, 2011). In order to use this system extensively, the optimal conditions must be determined for the highly reproducible VIGS phenotypes, as VIGS efficacy is affected by many environmental factors.

In this study, we optimized the conditions for the SYCMV-based VIGS system via the syringe infiltration in soybean. We investigated several factors that affect gene silencing efficiency in the soybean including photoperiod, plant age, culture concentration, and growth temperature (Fig. 2). Our results suggested that a LD (16/8 h) photoperiod, infiltration onto the cotyledons and primary leaves, an *Agrobacterium* culture OD_600_ of 2.0, and a growth temperature of approximately 27°C could promote gene silencing efficiency. Regarding temperature, soybean cyst nematode (SCN), bacterial leaf pustule (BLP), and rust bioassays are typically performed at 27°C, 28°C, and 25–30°C, respectively. Because a lower temperature (20°C) is optimal for the BPMV-based systems (Zhang et al., 2010), SCN and rust bioassays are conducted at 20°C (Kandoth et al., 2013; Meyer et al., 2009), but this cold treatment may inhibit nematode, bacterial, and fungal growth, as well as soybean growth. In contrast, we found that the optimal temperature of the SYCMV-based VIGS system was 27°C, which may be more effective in disease resistance research.

In addition, we confirmed that SYCMV-based VIGS could spread throughout the entire plants (Fig. 3). This finding may allow for the use of SYCMV-based VIGS to study abiotic stress tolerances in root tissues under flooding and drought conditions, disease resistance in leaves inoculated with bacteria or fungi, and gene function of vegetative and reproductive tissues such as stems, flowers, and pods.

Among three inoculation methods, agro-infiltration, particle bombardment, and direct plasmid DNA-rubbing in SYCMV-based VIGS system (Lim et al., 2015), we used the syringe infiltration method in this study, and the mechanical inoculation assay was additionally conducted because it is faster and easier than syringe for large-scale screening experiments. In order to determine whether mechanical transmission can occur from silenced plants to other plants in this VIGS system, SYCMV:*GmPDS*-infected tissues were mechanically inoculated with carborundum to healthy plants. We successfully obtained the same photobleaching results (Supplementary Fig. 3), implying that the SYCMV-based VIGS system could be effectively applied to assess a large number of plants using a soybean library.

In a screening experiment performed using sixteen soybean germplasms for an efficient SYCMV-based VIGS system (Fig. 4), we observed germplasm-dependent variations of photobleaching symptoms that varied from tiny white spots to more than 75% bleaching of the whole leaf area. Soybean cv. Jangyeobkong which was routinely used in our experiments and wild soybeans (GWS 0368, GWS 0999, and IT 182932) displayed the best performance in the SYCMV-based VIGS. Wild soybeans exhibited obvious photobleaching symptoms at 20 DAI, whereas cultivated soybeans showed the *GmPDS*-silencing phenotype at 25 DAI, and cv. SS2-2 displayed this silencing symptom at least 30 DAI. Notably, cultivar SS2-2 as a supernodulating mutant induces the overexpression of jasmonate-responsive genes such as *vspA*, *vspB*, and *Lox2* (Seo et al., 2007) and exhibited BLP resistance (Kim et al., 2008). Although SS2-2 containing a number of nucleotide-binding site-leucine-rich repeat (NBS-LRR) genes is a disease tolerant plant (Kang et al., 2012), gene silencing was successfully induced by increasing the concentration of the inoculum solution.

In conclusion, we optimized the protocol for an efficient SYCMV-based VIGS system via syringe infiltration. This system may serve as an easy-to-use tool for determining the functions of many candidate genes and for characterizing previously unidentified pathways associated with important agronomic traits.

## Supplementary Information
