# Supplementary material for: Optimization of a Virus-Induced Gene Silencing System with Soybean yellow common mosaic virus for Gene Function Studies in Soybeans
Source: Plant Pathol J. 2016 Apr 1;32(2):112–22. doi: 10.5423/PPJ.OA.04.2015.0063 (PMC4853101; doi:10.5423/PPJ.OA.04.2015.0063)
Supplement: Supplementary file 1 [file ppj-32-112-suppl.pdf]

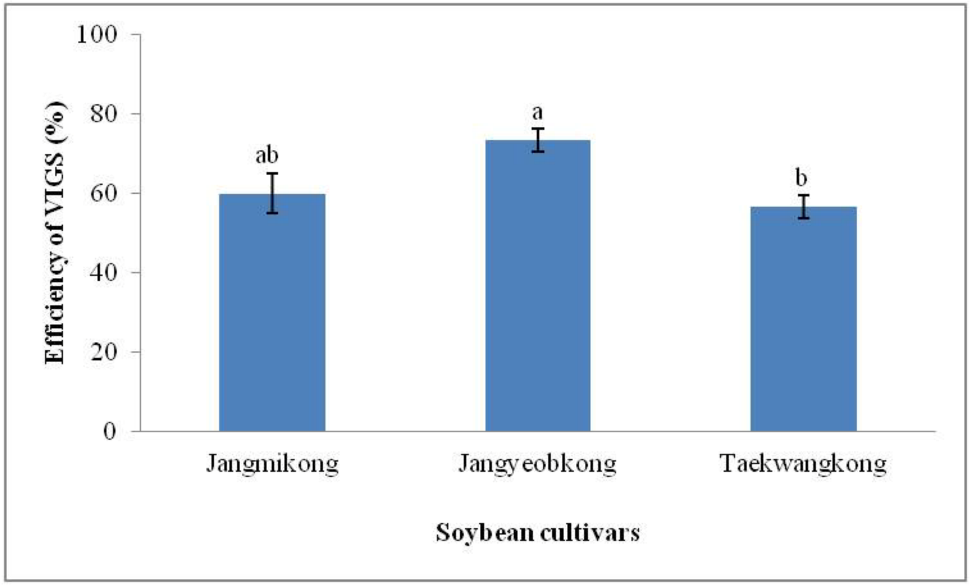

Supplementary figure 1. Effect of different cultivars for agroinoculated plants on SYCMV-based VIGS efficiency in soybean.

The SYCMV:*GmPDS* inoculum with OD_{600} = 0.8 were injected by the syringe-infiltration onto the primary leaves of three different cultivars, Jangmikong, Jangyeobkong, and Taekwangkong. These inoculated plants were grown in a green house under natural conditions. The photobleaching plants and efficiency of *GmPDS* silencing was analyzed after 30days after inoculation, and statistical analysis was described in material and methods.

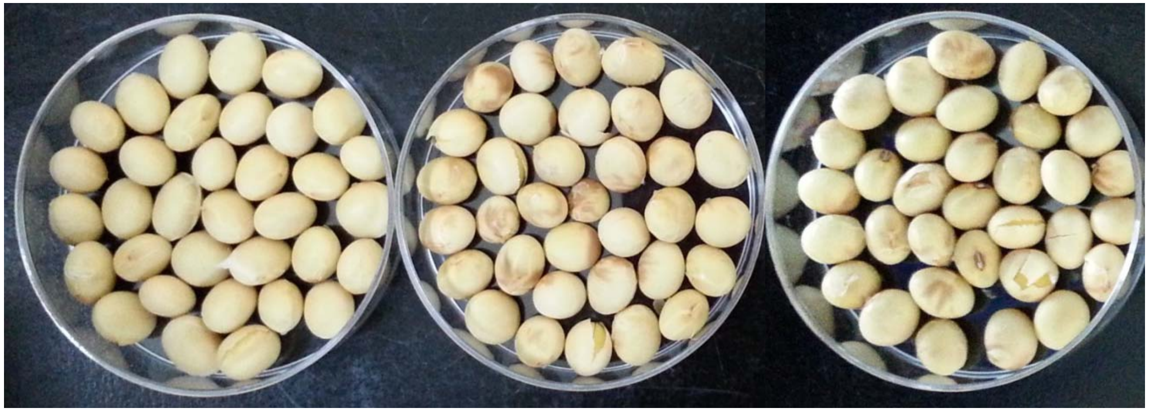

Mock SYCMV:empty SYCMV:*GmPDS*

Supplementary figure 2. Occurrence of seed coat mottling in soybean plants inoculated with SYCMV:empty and SYCMV:*GmPDS* constructs.

Seed coat mottling were evaluated at harvest after soybean plant infiltrated SYCMV:empty and SYCMV:*GmPDS* compared with healthy plants. All inoculated plants produced seed coat mottling, whereas non-inoculated plants showed no mottled seed.

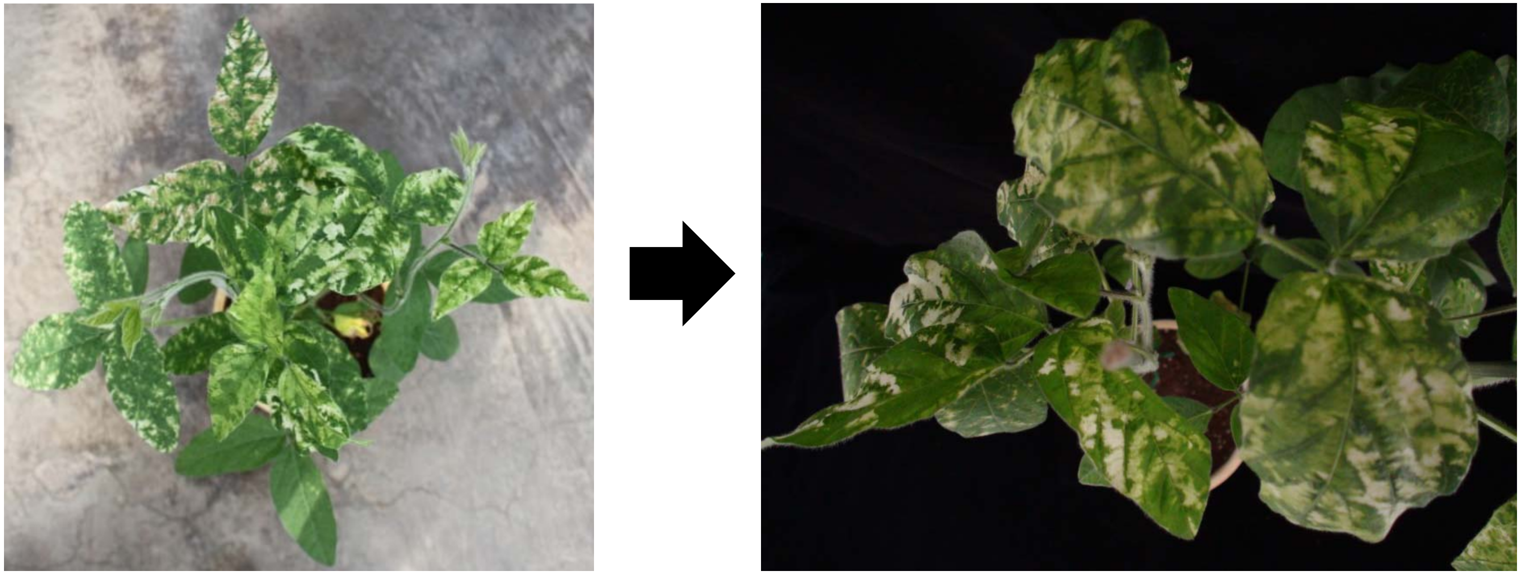

Supplementary figure 3. Photobleaching phenotype by mechanical inoculation with SYCMV:*GmPDS*-infiltrated leaves.

After performing phenotypic confirmations and *GmPDS* gene silencing in the SYCMV:*GmPDS*-infiltrated plants by syringe method, the infected leaves of the silenced plants were sampled. These leaves were used to mechanically inoculate the other plants. The mechanically inoculated plants showed successful photobleaching phenotype after 20-25 DAI.
